# Inequalities in the geographic access to delivery services in Brazil

**DOI:** 10.1186/s12913-024-12042-4

**Published:** 2024-12-18

**Authors:** Valdemar Pinho Neto, Cecilia Machado, Felipe Lima, Soraya Roman, Gilson Dutra

**Affiliations:** 1 Center for Empirical Studies in Economics, Fundação Getúlio Vargas, Rio de Janeiro, Brazil; 2 Graduate Program of Economics, Catholic University of Brasilia, Brasília, Brazil; 3 Department of Economics, Uppsala University, Uppsala, Sweden; 4 Department of Economics, PUC, Rio de Janeiro, Brazil; 5 FGV EPGE, Getulio Vargas Foundation, Rio de Janeiro, Brazil

**Keywords:** Access, Geo-referenced data, Health inequality, Childbirth

## Abstract

**Background:**

Despite Brazil’s recent social progress, access to health services is still unequal. This article analyzes the inter-municipal distances traveled by pregnant women to access delivery services, documenting their magnitude and relationship to socioeconomic and risk factors for over a decade in Brazil.

**Methods:**

Using data between 2007 and 2017 from the Brazilian Information System of Live Births and a matrix of inter-municipal distances, we describe the evolution of (i) the share of pregnant women that traveled across municipalities and (ii) the average distance they traveled. Next, we assess which of the previous variables explains the changes in travel distance over time. Finally, we estimate the difference in the average travel distance by individual risk factors and use regression analysis to measure the association between this distance and municipal socioeconomic determinants from the Brazilian census.

**Results:**

We observe that, on average, (i) the share of women traveling for childbirth increased, reaching 31% in 2017, and (ii) distances got longer, approaching the 60-kilometer mark by 2017. The increase in distance is mainly due to more women traveling. Nevertheless, regional disparities persist, especially between the north/inland and coastal regions. Women with high-risk pregnancies or newborns with risks such as low birth weight tend to travel longer distances. However, those residing in higher-development municipalities tend to travel shorter distances.

**Conclusion:**

Long distances remain an obstacle to accessing delivery facilities. This matter affects the most vulnerable disproportionately. Policymakers must consider the geographic accessibility of mothers when expanding birth-related services. Additionally, more research is required to understand the decision to travel and the distance effectively traveled as different accessibility facets.

## Background

According to the World Bank, health access is a fundamental pillar for life quality, and development [1]. Although 91.1% of the world population lives up to one hour from a hospital or clinic by motorized transport, only 56.7% are within this time limit by foot, and remote regions still need to be connected [2]. Concerning Brazil, despite many advances in poverty alleviation and inequality reduction over the last decades [3], access to public health services remains unequal. This inequality is evident when considering the distances people must travel to reach these services, as we will further demonstrate in this article, focusing specifically on delivery services.

Brazil’s Universal Healthcare System (SUS) has equal access as a guiding principle, but supply inputs differ from one region to another [4, 5]. For instance, workforce spatial distribution correlates with socioeconomic inequality, imposing challenges in guaranteeing a more balanced disposition of facilities and providers [6]. Besides that, it is also important to observe the adequacy to risk, looking into the qualitative aspects of care [7]. Previous research on obstetrics has shown space for improvement in this dimension: technology and practices also vary geographically and correlate to mother’s characteristics [8]. Only half of the public health services assisting at-risk newborns have a NICU (Neonatal Intensive Care Unit), while private hospitals with NICUs primarily provide care to low obstetric-risk newborns [9]. Therefore, a more suitable match between the case’s needs and facilities should be offered [10], especially considering the delays in healthcare search [11].

According to data analysis based on Birth in Brazil (*“Nascer no Brasil”*) questionnaire, collected in 2011 and 2012, 98.7% pregnant women had some prenatal assistance, 75.8% initiated it before the sixteenth gestational week, and 73.1% had six or more prenatal appointments during pregnancy. Nonetheless, only 58.7% were directed to a reference maternity unit during appointments, and 16.2% had gone to alternative facilities prior to delivery [12]. Hence, although coverage for prenatal care is elevated in Brazil, few women have received proper guidance on where to go for childbirth.

In 2007, the median distance Brazilian mothers traveled across municipalities to give birth was 21 km, with the North region surpassing 33 km [13]. Over the last decades, the North and Northeast were “hotspots” for neonatal and maternal mortality and comprehensive emergency newborn and obstetric care can be more than two hours away in several areas of those regions depending on the specific equipment [14]. For instance, distances for pediatric ICU are more than 120 kilometers away for more than 30 million people [15].

Additionally, traveling has been associated with worse outcomes for newborns [16] and mothers [17]. Similar associations and causality have been shown in Portugal, Australia and The Netherlands [18–20], while difficulties in access are somewhat ubiquitous [2, 21–25]. Another study also found a positive association between the distance to delivery facilities and the infant mortality rate for the Brazilian case [13].

This article aims to comprehensively describe the trends and regional inequalities in the distances pregnant women traveled for their childbirth between 2007 and 2017. In this circumstance, they could choose to travel in search of better health services or, in many cases, to access the hospital closest to the municipality in which they reside due to the lack of availability of maternity wards in all Brazilian municipalities. Our analysis is based on a novel decomposition exercise that focuses on two measures of geographic accessibility: (i) the share of pregnant women traveling to give birth and (ii) the distance these women traveled, calculated at the state-level. Moreover, we identify the relationship between distances to delivery facilities and socioeconomic and risk factors. Our paper expands the findings of previous studies by analyzing a longer time span combined with multiple cross-section comparisons. Lastly, we are able to look into the association between geographic access and birth-specific characteristics, such as the mother’s socioeconomic background and the mother and baby’s health risk factors.

## Methods

### Scope

This study is one of three articles within a larger project aiming to explore the impact of physical distances to childbirth facilities on neonatal and maternal outcomes in Brazil between 2007 and 2017^1^. In this article, we offer a comprehensive overview of the movement of pregnant women to childbirth facilities from a demand-side perspective. Specifically, we examine the average individual characteristics (i.e., risk factors) and social determinants to understand their migration patterns. The second article details the changes in the availability of health services from a supply-side perspective, while the final article explores the causal pathways that may clarify the effect of distances to childbirth facilities on neonatal mortality. The topics of the second article are not detailed here, which constitutes a limitation as some of the changes in pregnant women’s movement patterns may be explained by changes in the availability of health facilities. We address this alternative explanation by referencing the findings of our other research in the Discussion section.

Our analysis consists of three parts. First, we identify and analyze the inter-municipal distances pregnant women travel to receive health attention during childbirth, dividing the analysis between the share of women traveling and the distance they traveled. We aggregate our estimates at state and national levels to identify geographic and time trends. Then, using individual administrative records, we analyze the relation between the traveled distance and risk factors. Finally, we use census data to conduct a regression analysis of the association between social determinants and the average distance. We seek to provide a broad description of movements by pregnant women giving birth and move to an analysis of their characteristic to better identify who they are on average, suggesting reasons for the patterns in this observed migration.

### Data sources

We employ the Brazilian Information System of Live Births (Sistema de Informações sobre Nascidos Vivos - SINASC) to identify pregnant women’s residence and destination for childbirth, and additional information that characterizes their socioeconomic background and particularities of their medical cases, such as pregnancy risk and birth outcomes (e.g., birth weight, prematurity). The database consists of administrative records from DATASUS (Health Ministry database), compiled, treated, and enriched by the Data Science Platform applied to Health (Plataforma de Ciência de Dados aplicada á Saúde - PCDaS) using ETL methodology [26].

The inter-municipal distances were obtained from CEDEPLAR-UFMG. Using Google Maps APIs and OpenStreet Maps, CEDEPLAR-UFMG estimates the distances in kilometers and travel time between the 5,570 Brazilian municipalities. The output is a dataset containing distances among all possible origin-destination combinations [27, 28]. We use each pregnant woman’s residence and newborn’s birthplace to identify the corresponding distance from the CEDEPLAR-UFMG database and include it in the SINASC database.

Moreover, we complement our dataset with socioeconomic variables extracted from the 2010 Brazilian Census, in order to describe social determinants and traveled distances relationship [29] and bypass quality issues of such information contained in SINASC.

### Analysis of geographic patterns

We employ a simple model to decompose and weigh properly, which drives the current portrait of pregnant women traveling for childbirth. Three variables were constructed, *D*, *F* and *C*: *“D”* represents the average distance traveled by *all* pregnant women in a given municipality (travelers and non-travelers); *“F”* is the fraction of those women who traveled^2^; and *“C”* is the average distance, conditional on having traveled^3^ (i.e., restricted to positive inter-municipal distances).

First, we focus on *F*, the share of travelers, which compares the births outside the mother’s residence municipality with respect to all births in that municipality. Moreover, identifying travelers and non-travelers allows us to depict the flow of pregnant women between municipalities. Then, we focus on the conditional distance, *C*. In both cases, we aggregate the data at state and national levels to illustrate tendencies over time and by geographic zones. The aggregated indicators are weighted by the number of births per municipality. Additionally, we estimate the Gini index for the conditional distance using *ineq* R package.

Finally, we estimate the part of the 2017-2007 change in the average distance attributed to changes in the share of women traveling and the part attributed to changes in the average length of the travels. Our variables relate in the following way: $D=F\times C$. Hence, given two years *t*0 e *t*1, we can break the time average variation in the distance, *D*, into two components, shown in Eq. 1 below. $\Delta F\times C_{t0}$ represents more people traveling over the years and $F_{t0}\times \Delta C$ signals that average distances are increasing with time for travelers. Symbol $\Delta$ stands for difference between periods, as in $\Delta F = F_{t1} - F_{t0}$.

1 $$\begin{aligned} \Delta D \approx \Delta F\times C_{t0}+F_{t0}\times \Delta C \end{aligned}$$

### Individual characteristics analysis

In order to evaluate which characteristics of pregnant women and their babies are related to the travel distance, we split the sample by whether a characteristic/risk factor was present or not and estimated the average distance traveled (*D*) by each group. Then, we estimate the differences in means between the two groups (between the group of pregnant women with the characteristics/risk factor and those who did not have the characteristics/risk factor). Pregnant women and babies’ characteristics come from the SINASC database; they are newborn’s APGAR score lower than 8^4^, multiple pregnancies, low birth weight, prematurity (less than 37 gestational weeks), at least one pregnancy risk (congenital anomaly, multiple pregnancy, prematurity, or mother at risk age), secondary education or more (high school), cesarean, and more than seven prenatal controls.

### Social determinants analysis

We analyze the relationship between municipal socioeconomic measures and the average distance traveled by pregnant women for childbirth. We estimate the following regression model:

2 $$\begin{aligned} D_{i} = \beta _{0} + \beta _{1}X_{i} + \epsilon _{i} \end{aligned}$$

In which: $D_{i}$ is the average traveled distance made by pregnant women of a municipality *i*, same as in Eq. 1; $X_{i}$ is a socioeconomic variable; and $\epsilon _{i}$ is the error term, encompassing all omitted explanatory variables. The model is estimated using one socioeconomic variable at a time. The $X_{i}$ variables were standardized to z-scores for comparability ($X^z_i=\frac{X_{i}-\bar{X}}{sd(X)}$). Therefore, one should interpret $\beta _{1}$ as the additional distance associated with one extra standard deviation of *X*. We discuss the results and limitations of this model in the next section.

## Results

### Geographic patterns

**Fig. 1:**
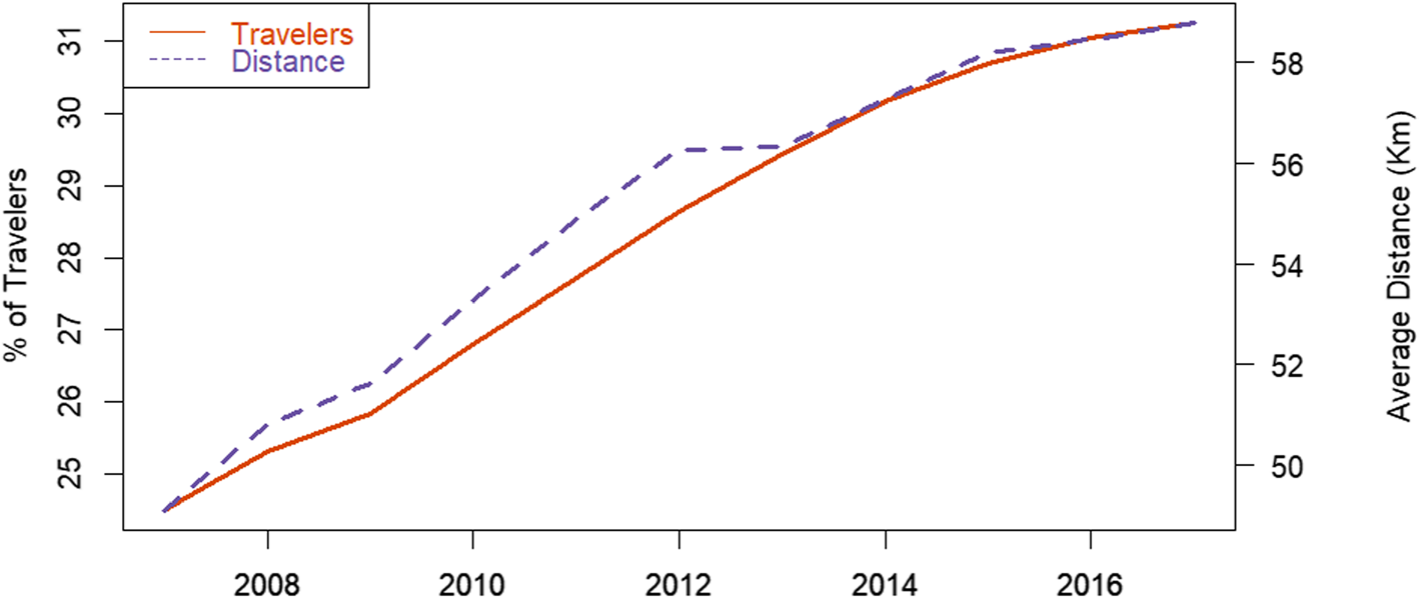
Share of pregnant women traveling and distance. The figure shows the share of pregnant women moving to another municipality to give birth (LHS - in %) and the average distance conditional on traveling (RHS - in kilometers) at the national level, ranging from 2007 to 2017

Figure 1 illustrates the shifts in national travel patterns over the last few years in Brazil. Both the share of pregnant women traveling between municipalities and the average distance they travel to access health facilities for birth were lower in 2007 compared to 2017. The share of traveling women went up from 24% in 2007 to 31% in 2017, while the average distance rose from about 48 to 59 kilometers. During this period, these indicators’ growth has been steady and almost linear, excepting 2012–2013 when distances were kept constant but resumed escalating right after.

Existing evidence indicates that similar patterns are observed for total hospitalizations and hospitalizations due to various causes other than births. The share of people moving to another municipality or health region for hospital care has significantly increased in Brazil, particularly over the last decades [31]. This paper highlights a broader trend of regionalization of hospital care, providing a better understanding of the dynamics influencing hospital admissions in Brazil, with a specific focus on hospitalizations for births.

Next, we describe the patterns of the share of pregnant women traveling for childbirth by municipality and state. A description of the conditional average distance traveled by these women by state over time follows the previous analysis. Finally, we join both variables to identify how and which of them explains the variations in the unconditional traveled distance, using the decomposition model presented in the methods section.

#### Share of pregnant women traveling

**Fig. 2:**
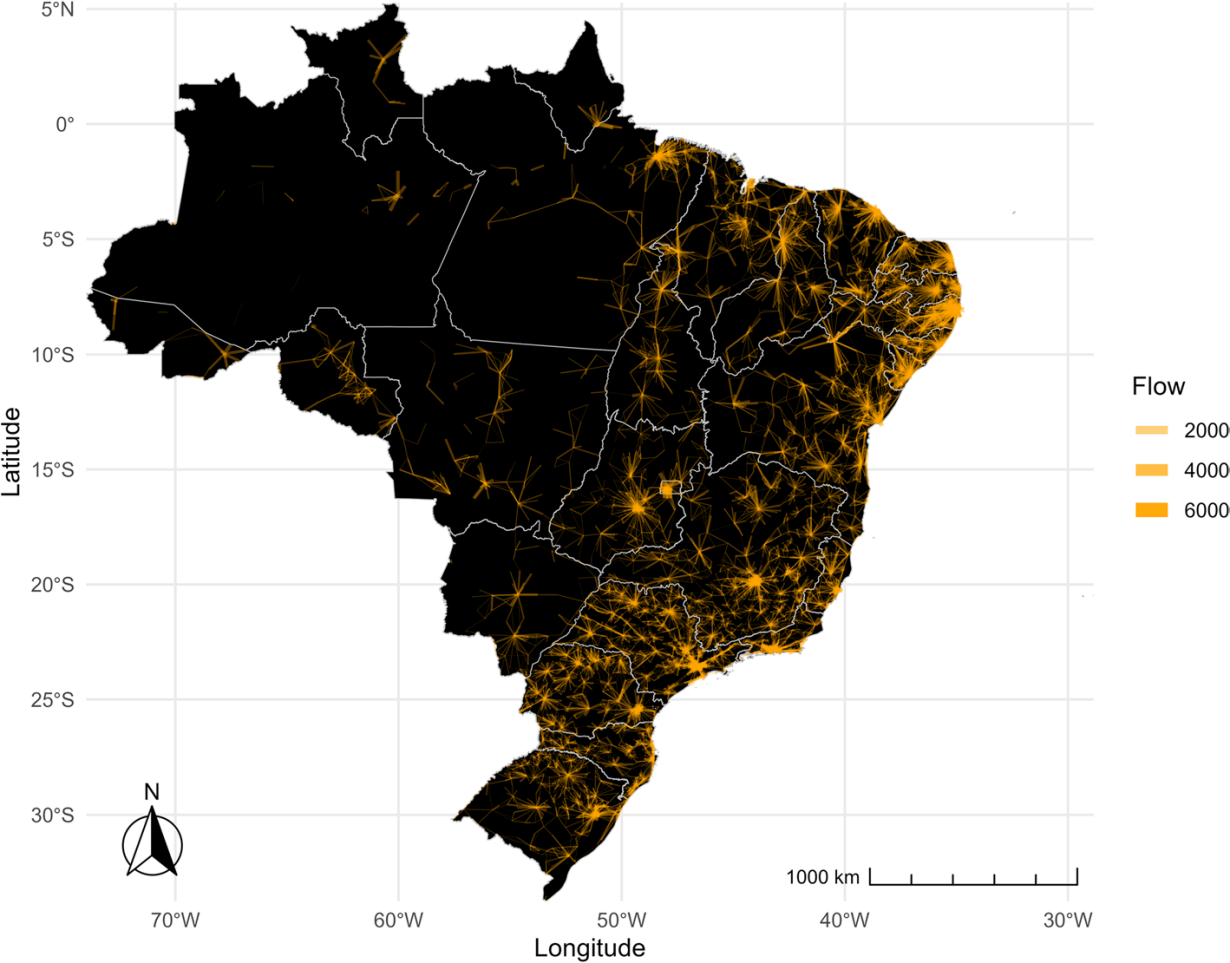
Intensity of moving flows. The lines in the figure show the connections between the municipalities of residence and destination. The thicker the lines, the higher the share of women moving between municipalities

**Fig. 3:**
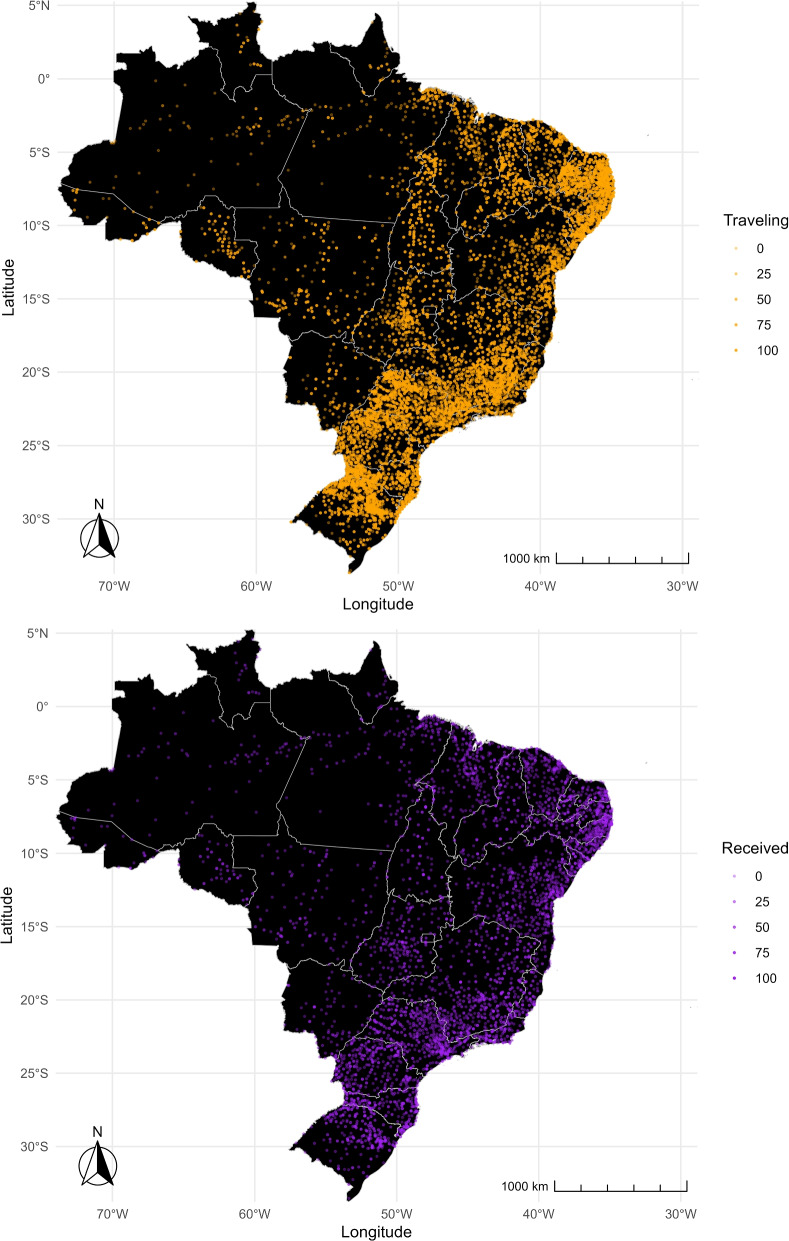
Departure and arrival. Orange dots show the percentage of departures, representing the proportion of pregnant women leaving their municipality of residence for childbirth (top map). Purple dots show the percentage of arrivals, representing the share of pregnant women coming to a municipality from other municipalities for childbirth (bottom map). The color of the dots becomes more transparent as the shares become smaller

Disparities arise across Brazil, as shown in Figs. 2 and 3. In the former, the orange lines depict the flows of pregnant women between municipalities, with the thickness of the lines indicating the volume of such travels. The figure reveals a star-like pattern of flows, with a central municipality serving as a hub for the surrounding areas. The distance between municipalities and the thickness of the flows vary significantly across regions. Travel volumes are notably higher, and distances between municipalities are shorter in the South, Southeast, and Northeast regions compared to the North and Central-west regions. Moreover, while there are distinctive focal points-municipalities where most flows converge- in the eastern part of the country, in the North and West, flows are more dispersed across multiple municipalities, lacking a central concentration point.

Figure 3 complements Fig. 2 by illustrating the percentage of pregnant women leaving their municipality for childbirth (departures) and the percentage of pregnant women arriving in a municipality from other municipalities for childbirth (arrivals). The top map displays orange dots representing the proportion of pregnant women leaving their municipality, whereas the bottom map shows purple dots representing the proportion of pregnant women arriving in a municipality from other areas. In both maps, a more solid color indicates a higher percentage of pregnant women. The South, Southeast, and Northeast regions show solid purple and orange dots, suggesting that these areas send and receive pregnant women for childbirth. Further, municipalities lay close to each other over short distances, mixing departure (solid orange) and arrival (solid purple) cities and suggesting more frequent but shorter travels most likely contained within the regions. In contrast, the North region displays more transparent purple and orange dots, indicating a lower percentage of departures and arrivals within the region. Unlike other regions, there are no apparent sender and receiver municipalities. Hence, if pregnant women in the North leave their municipality for childbirth, they are more likely to travel longer distances to reach municipalities in other regions. Subsequent sections of the paper will present further evidence supporting these findings.

**Fig. 4:**
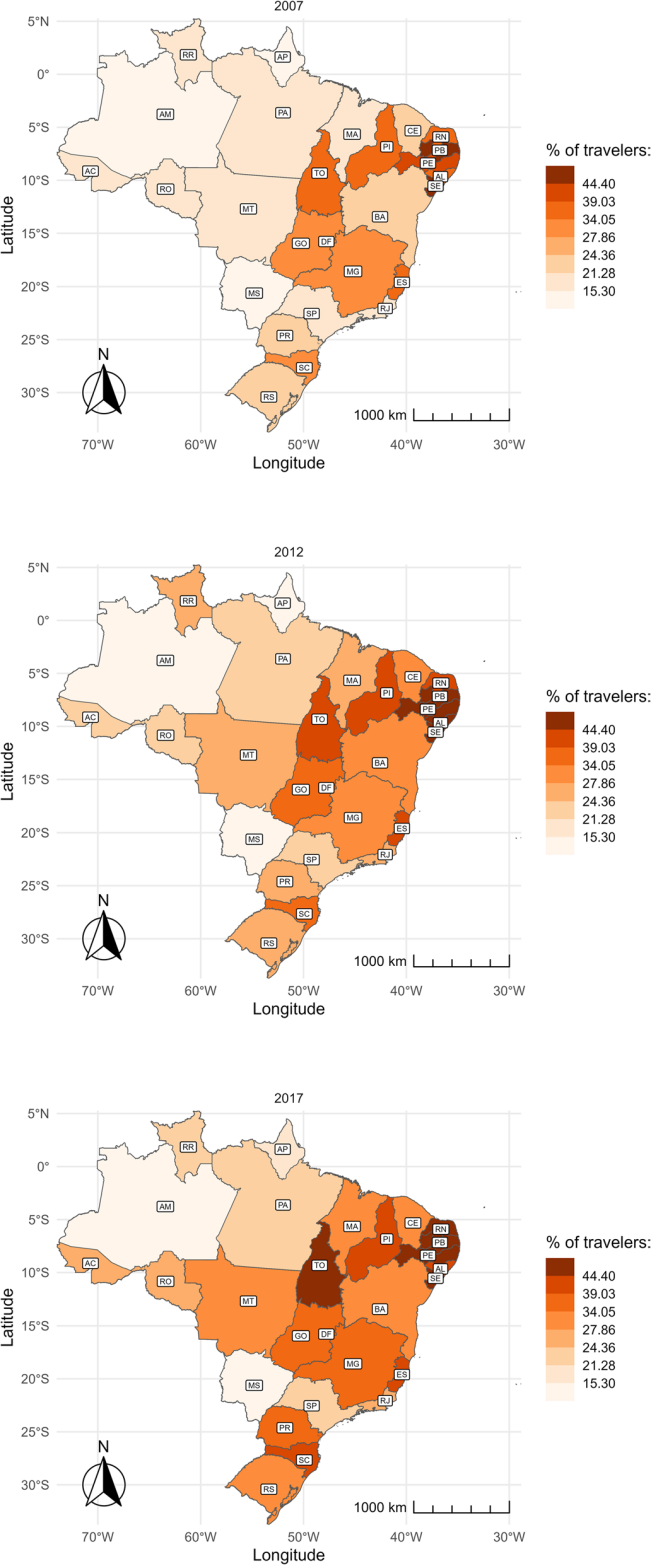
Share of travelers over time and by state. The figure shows the share of women who traveled to another municipality to give birth by state in 2007 (top), 2012 (center), and 2017 (bottom). The abbreviation of the name of the States is represented in Table 3 in the Appendix

The following maps show the share of childbirths that happen outside of a mother’s residence municipality, as opposed to the share of those that take place in the same municipality where she lives. Hence, we are able to visualize places where pregnant women often leave searching for delivery services. Figure 4 depicts which states have the highest traveling shares. Colors were defined based on the percentiles of nationwide shares, divided into eight brackets of 12.5 percentiles each. Consistent with Figs. 2 and 3, we find that the share of pregnant women leaving their residence municipality to give birth is higher in the Northeast and Southeast regions than in the other regions. Across the years, we verify that the vast majority of states had an upsurge in such movements. Five of them have surpassed 44% by 2017, placing in the top bracket: Sergipe (SE), Pernambuco (PE), Paraíba (PB), Rio Grande do Norte (RN) e Tocantins (TO).

**Fig. 5:**
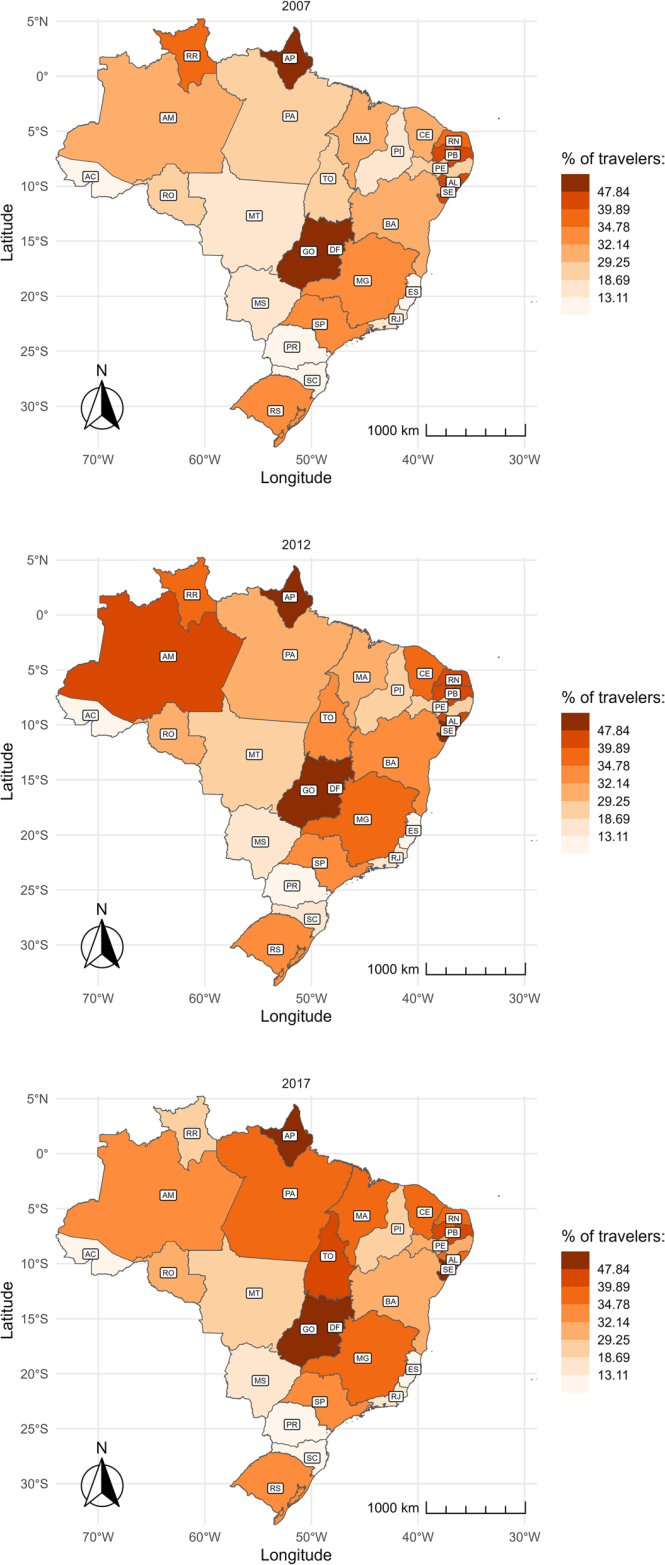
Share of travelers leaving their health region over time and by state. Health regions are local networks of facilities that include more than one municipality. The figure shows the share of women who moved outside their health region to give birth by state in 2007 (top), 2012 (center), and 2017 (bottom). The abbreviation of the name of the States is represented in Table 3 in the Appendix

Using the sample of pregnant women who travel to give birth, we analyze the percentage which leave their health region. Figure 5 displays this percentage by state. In northern states, the percentage of travelers leaving their health region for childbirth is higher than the percentage of pregnant women who travel (Fig. 4). This means that, although traveling is uncommon, the likelihood of leaving the health region when the mother travels is high in these states. In contrast, southern states have a high percentage of pregnant women who travel for childbirth but a small percentage of those leaving their health region.

Over the decade, more pregnant women are traveling outside their health region to give birth. The states of Amapá (AP), Goiás (GO), Alagoas (AL), and Distrito Federal (DF) have the highest rates, with over 47% leaving their original health region for childbirth. This tendency is less prominent in the southern states.

#### Average conditional distance

We focus on the sample of pregnant women that left their municipality of residence for childbirth and estimate the average distance they traveled (conditional distance). The evolution of this distance by state is presented through color maps. As in Fig. 4, colors were defined based on the percentiles of nationwide averages, divided into eight brackets of 12.5 percentiles each.

**Fig. 6:**
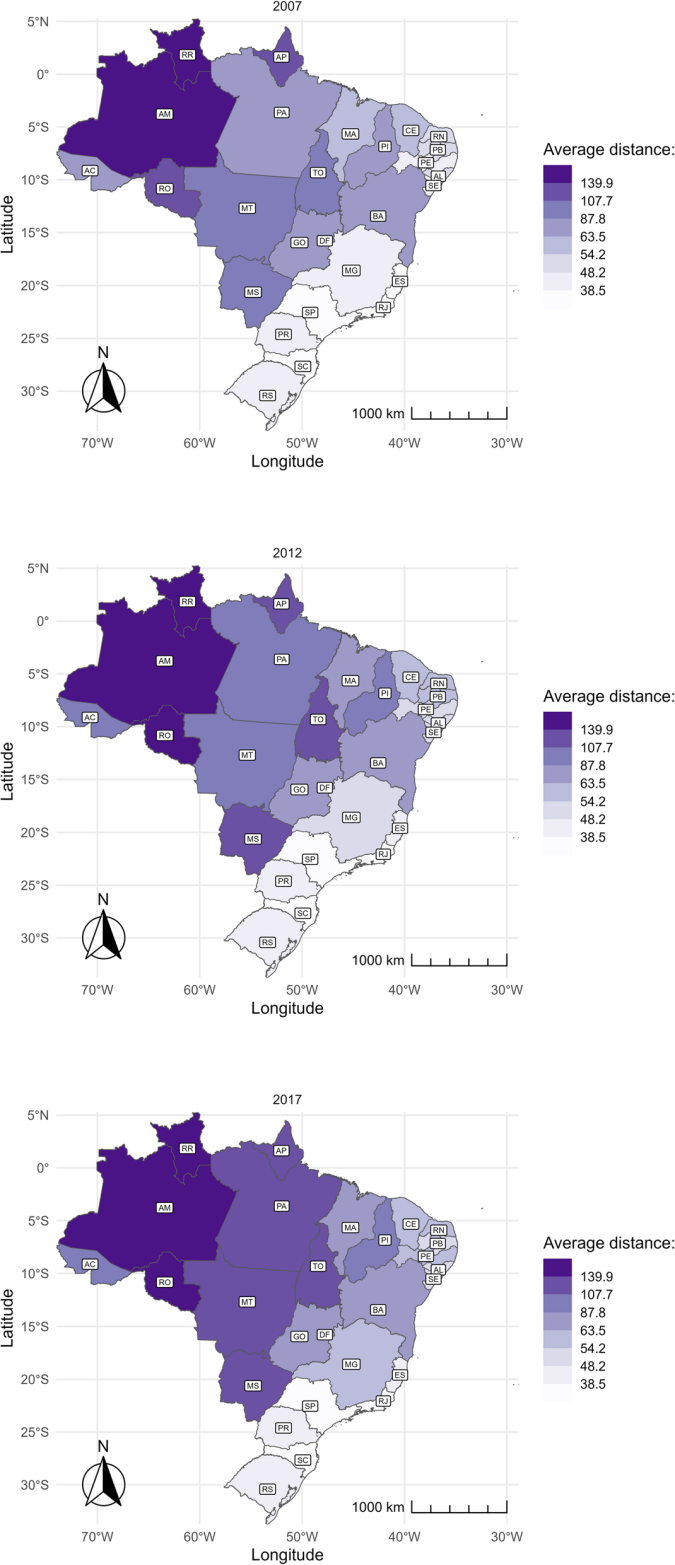
Average distance of travelers over time and by state. The figure shows the average distance traveled by pregnant women who moved to another municipality to give birth by state in 2007 (top), 2012 (center), and 2017 (bottom). The distance is measured in kilometers. The abbreviation of the name of the States is represented in Table 3 in the Appendix

Figure 6 shows that pregnant women in the North and Central-West regions travel the farthest distances to give birth, measured in kilometers. Additionally, these distances have increased over time, as indicated by the darker shading on the map while moving from 2007 to 2017. In 2017, even though the percentage of travelers was low, the average distance covered by these women was over 108 km, which falls into the top two brackets. On the other hand, women in the southeast travel the shortest distance, with a maximum of 38.5 km.

**Fig. 7:**
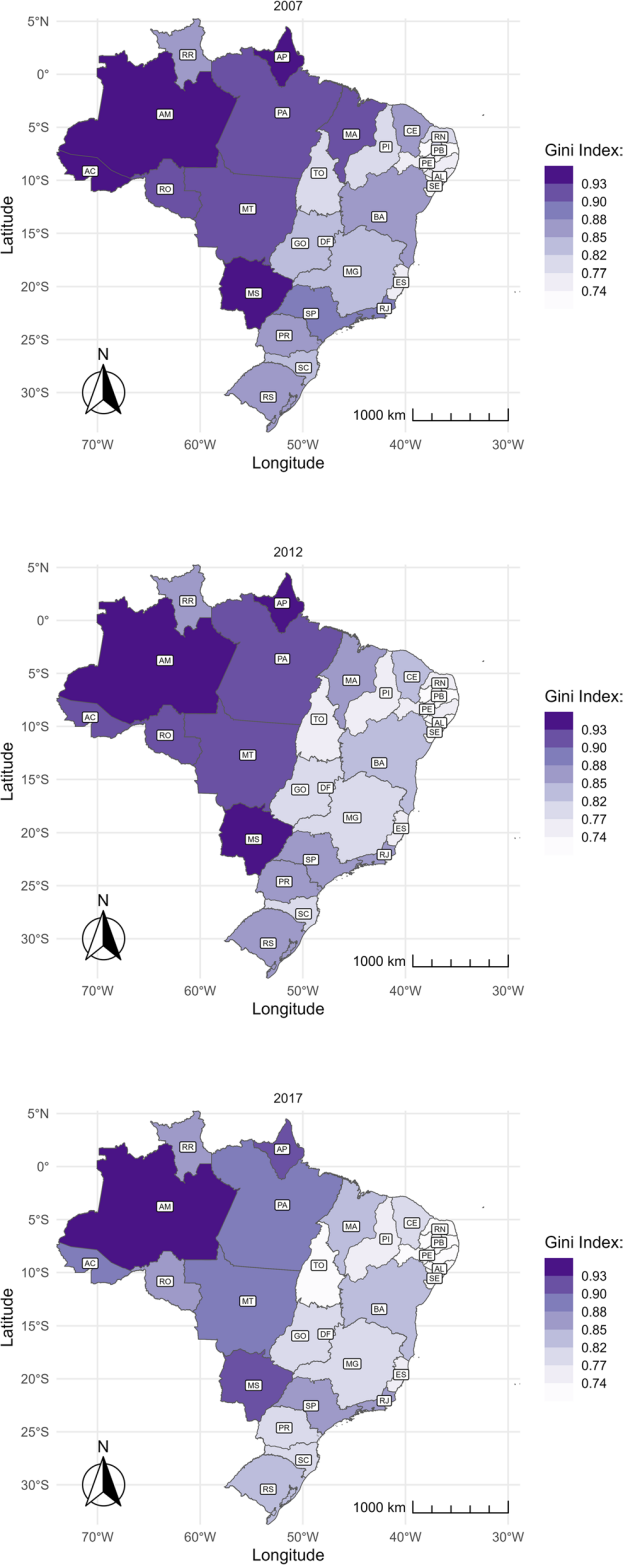
Gini coefficient of the municipal average distance of travelers over time and by state. The figure shows the Gini index of the average municipal distance conditional on traveling to give birth in 2007 (top), 2012 (center), and 2017 (bottom). The abbreviation of the name of the States is represented in Table 3 in the Appendix

To better understand the geographic distribution and concentration, we assessed the Gini index for traveled distances within a state. Figure 7 reveals: i) a greater inequality located in regions North and Central-West; and ii) inequality presents a downward trend, reducing in most states between 2007 and 2017.

#### Decomposition of 2007–2017 distance changes

**Fig. 8:**
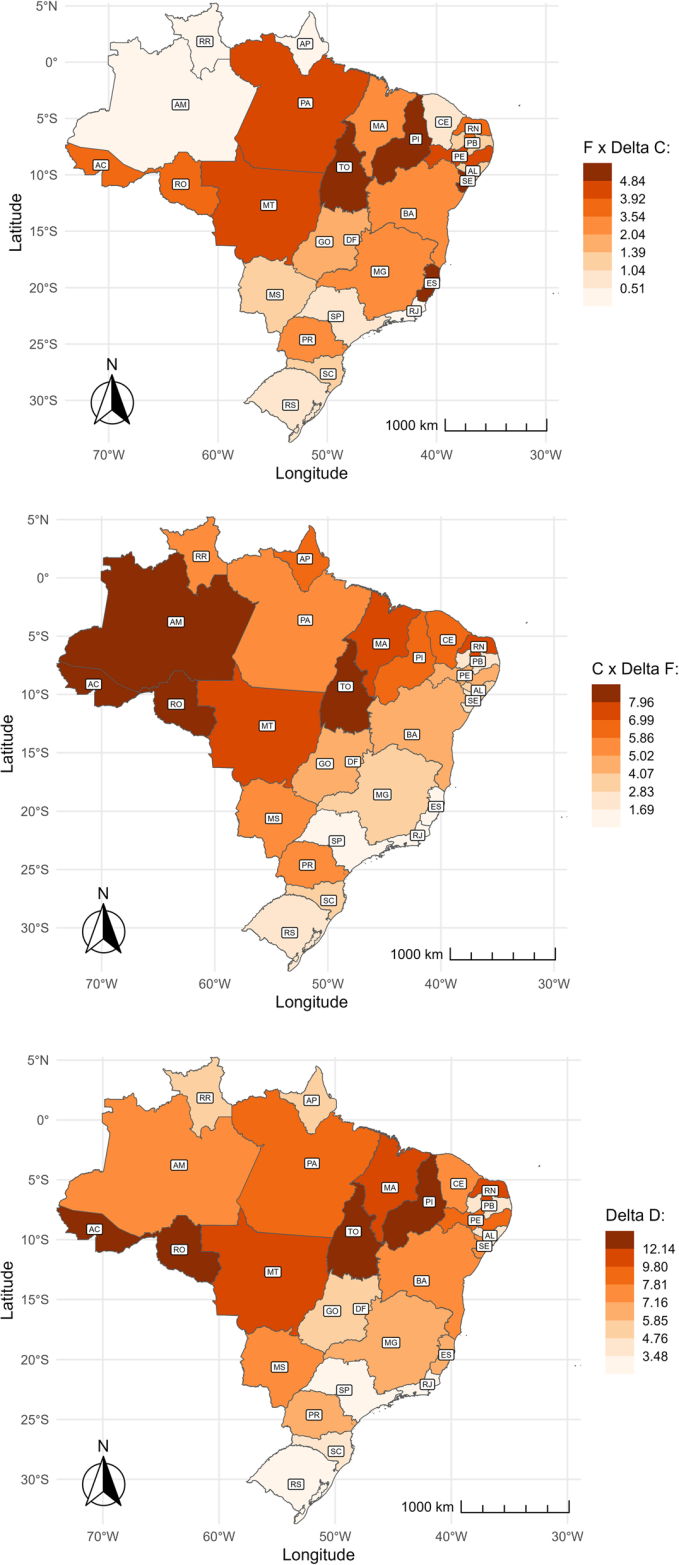
Decomposition of shares and distance over time and by state. $\Delta C \times F$: Changes attributed to trip length (top). $\Delta F \times C$: Changes attributed to the share of travelers (middle). $\Delta D$: Changes in the unconditional distance (bottom). The abbreviation of the name of the States is represented in Table 3 in the Appendix

Our research has shown that the number of pregnant women who leave their municipalities to give birth has increased between 2007 and 2017, along with the average distance they travel. As a result, we anticipate an overall increase in average distance. This section shows how much of this increase can be attributed to more pregnant women leaving their municipalities versus the increase in the number of kilometers they are traveling. Figure 8 displays the changes in the average distance traveled to the childbirth place by state, decomposed using Eq. 1. At the top, we present the part attributed to changes in the length of the trip; in the middle, the part related to changes in the share of pregnant women traveling; and at the bottom, the total change.

The map at the bottom of Fig. 8 shows that the average travel distance varied across states. The Central-west, North, and Northeast regions experienced the most significant increase, while the South and Southeast regions had the smallest increase. In the North and Northeast, states such as Tocantins (TO), Acre (AC), Rondônia (RO), and Piauí (PI) had the most significant increase, with a gain of over 12 kilometers. In the South and Southeast regions, São Paulo (SP), Rio de Janeiro (RJ), and Rio Grande do Sul (RS) had the lowest overall variation, remaining relatively stable.

The average travel distance for childbirth increased mainly due to more women traveling instead of the length of their trip. In Fig. 8, the top map shows that the median change attributed to the length of the trip is 2.04 km, while the middle map indicates that the median change due to the share of travelers is 5.02 km. The variation in effects differs by state and region. Changes in Amazonas (AM), Roraima (RR), Amapá (AP), and Ceará (CE) in the North and Northeast regions are almost entirely explained by changes in the share of travelers. On the other hand, in several states in the Southeast and coastal Northeast, the change in the average travel distance is attributed almost equally to an increase in the number of women traveling and the length of their trip. For example, in Minas Gerais (MG) and Bahia (BA), the increase in distance due to more women traveling ranges from 2.83 to 5.02 km, while the increase due to the length of their trip ranges from 3.54 to 3.92 km.

The geographic patterns of the distance traveled by pregnant women for childbirth described so far are also related to the size of a municipality, measured by the number of births each year. It is more frequent to observe pregnant women leaving their municipalities to give birth if their municipalities are small. As Table 1 shows, in municipalities with less than 35 births per year, 92% of pregnant women traveled for childbirth in 2007 and 96.3% in 2017. Conversely, in municipalities with more than 852 births per year, only 14 and 18% of pregnant women traveled for childbirth. In addition, consistent with the previously presented evidence, the average distance traveled among this group is shorter in small compared to large municipalities. These statistics are congruent with the organization of health attention in networks, usually based on population criteria: Instead of having a delivery facility in each municipality, small municipalities refer pregnant women to larger cities where they are expected to receive more comprehensive care.

The increase in the share of pregnant women traveling for childbirth and their distance happened across all municipality sizes. Nevertheless, the relative increase in the average conditional distance was greater for small municipalities (deciles 1 to 3) than for medium to large municipalities (deciles 6 to 9). For instance, the distance increased from 46 to 56 km, a 25% rise, for municipalities with less than 35 births per year. In contrast, in municipalities with 150 to 208 births per year, the distance increased by approximately 4 km, a 5% increase. On the other hand, the changes in the proportion of pregnant women traveling mainly affected medium to large municipalities instead of small municipalities. In the Discussion section, we suggest that these changes in pregnant women’s mobility patterns could be related to the regionalization process of the health system.

**Table 1:** Average share of travelers and conditional distance by municipal number of births in 2007 and 2017. The Deciles column contains the decile of the yearly number of births per municipality over the analysis period. The Interval columns show the range of births per decile. The Share of travelers is the percentage of pregnant women who traveled outside their municipality of residence for childbirth, and the Average conditional distance is the average distance among the pregnant women who traveled

Deciles	Interval		Share of travelers		Average conditional distance	
	Min	Max	2007	2017	2007	2017
1	1	35	92.0	97.7	46.5	59.0
2	36	55	85.8	95.0	55.2	65.6
3	56	78	82.4	93.7	55.0	62.4
4	79	108	71.0	88.9	63.5	67.2
5	109	149	68.1	82.8	62.2	71.5
6	150	208	55.5	74.0	72.9	76.4
7	209	294	45.1	63.3	80.7	84.2
8	295	445	37.2	54.1	90.5	98.5
9	446	852	24.6	37.5	107.4	118.5
10	853	176487	14.6	17.7	130.6	159.0
Total	1	176487	24.5	31.3	115.6	137.8

### Distance and mother-child risk profile

**Fig. 9:**
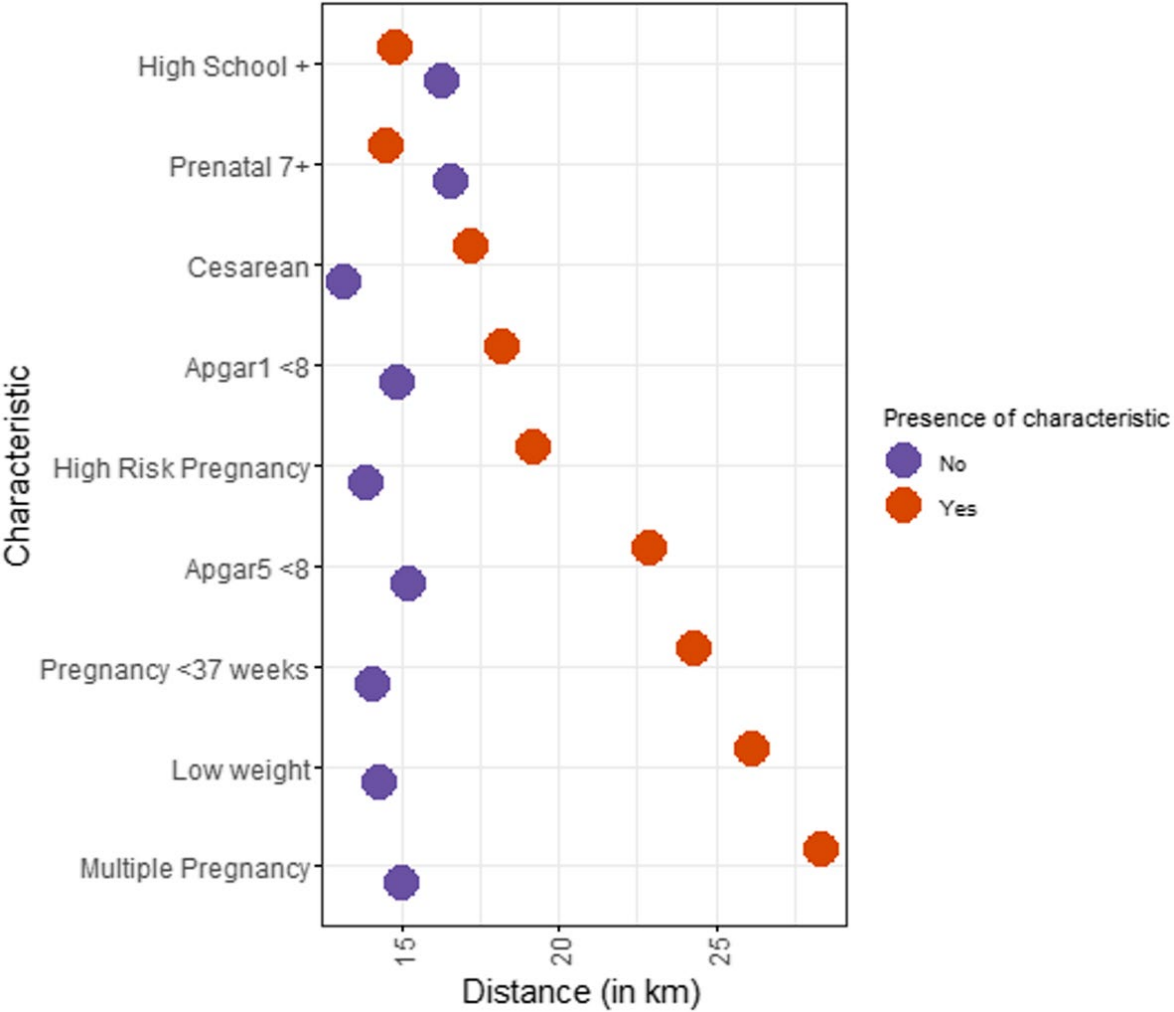
Individual characteristics/risk factors and average traveling distance. The figure shows the average distance pregnant women travel to give birth (x-axis) by the presence or absence of a risk factor (y-axis, Yes in orange and No in purple)

Figure 9 shows the average travel distance for mothers and babies with and without a given characteristic or risk factor, marked by orange and purple circles, respectively. Most distinctive gaps in traveled distances are indicated by risk-related factors. Namely, pregnancies with less than 37 weeks, multiple pregnancies, high-risk pregnancies, low-weight fetuses, and low APGAR scores. On average, pregnant women with any of the previous risk factors travel more for childbirth than other pregnant women. Having a c-section during labor is also correlated with longer traveling distances, but the difference in distance between having and not having a c-section is smaller. The difference in the average distance by education, divided into at least high school and less than high school, is even smaller. Likewise, on average, pregnant women who attend at least seven prenatal appointments travel a similar distance as those who attend fewer appointments.

### Socioeconomic background and its relationship with distances

**Fig. 10:**
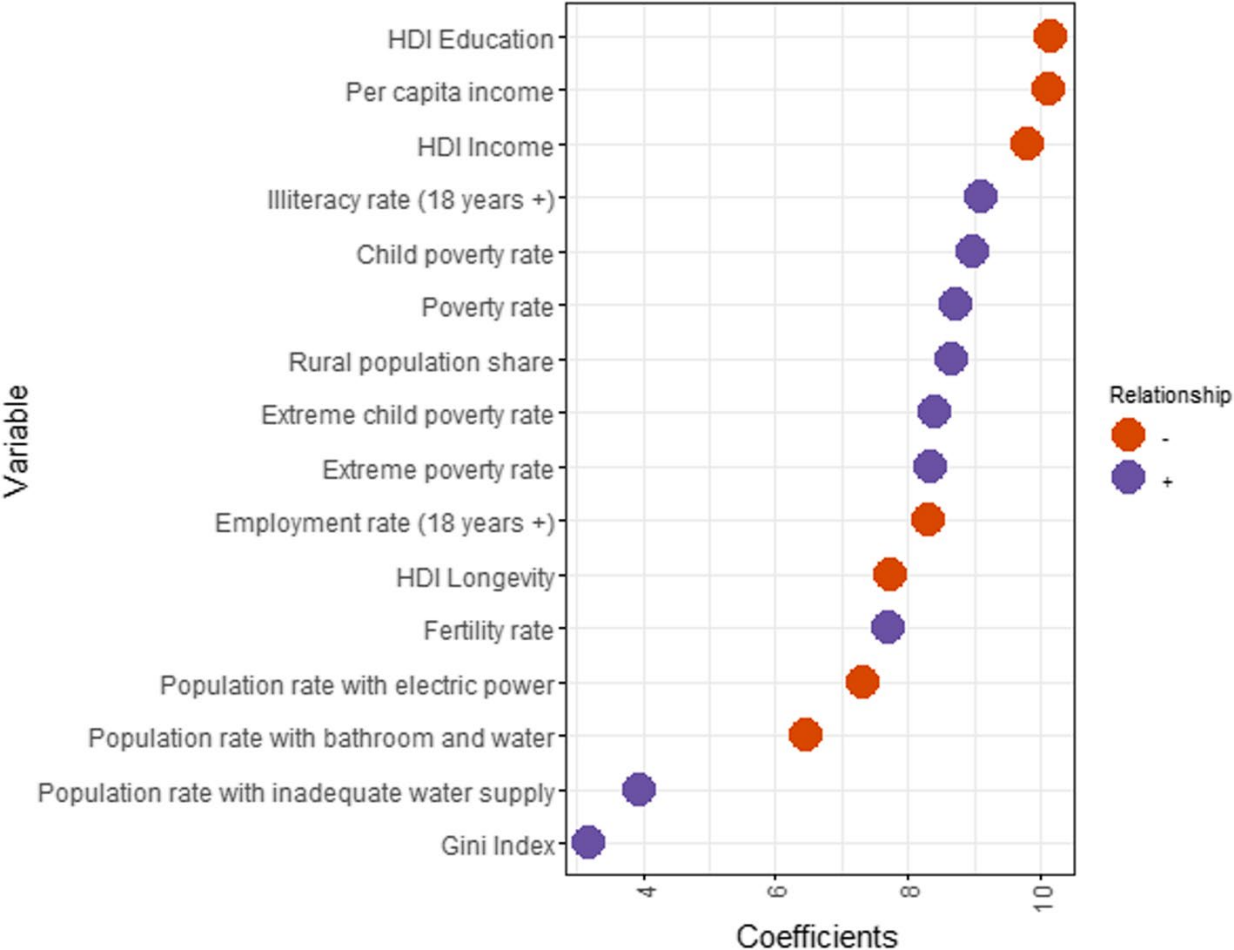
Socioeconomic factors and distance. The figure shows estimated coefficient magnitudes (x-axis) of each socioeconomic factor (list on y-axis) regressed separately at the municipal-level against average travel distance. Colors indicate the correlation signal: positive in purple and negative in orange

Figure 10 displays municipal socioeconomic variables on the y-axis and the magnitude of Eq. 2’s $\beta _{1}$ coefficient on the x-axis, which measures the partial correlation between the socioeconomic variables and the traveled distance. Positive coefficients are marked in orange, while negative ones are in purple. Variables with negative association are, for instance, per capita income, HDI education, and HDI income. This means pregnant women living in municipalities with higher scores on these measures are expected to travel fewer kilometers. On the other hand, the child poverty rate, illiteracy rate, and population poverty rate are positively associated with the traveled distance. Overall, the results are intuitive and coherent, as distances are expected to be shorter in areas with higher development levels. Univariate regressions are not controlling for the other variables on the list, but show that any of the used measures leads to the same conclusions^5^.

## Discussion

This study examines how geography affects access to delivery services by analyzing two dimensions of the distance to health facilities: the share of pregnant women traveling and the average distance they travel. The patterns of these factors are opposites. When the travel distance is long, fewer women leave their municipality for childbirth, but the frequency of travel increases if it is short. Consistent with this finding, evidence from developing countries shows that the likelihood of having an institutional delivery decreases as the distance to health facilities for childbirth increases [22, 32]. If the average distance had been considered by itself, these differentiated patterns would have been overlooked, and regional differences would have been less noticeable.

The share of travelers and the distance traveled for childbirth vary significantly between coastal regions and northern/inland regions. This difference is closely related to the availability of healthcare facilities. Emergency neonatal care is unavailable within the two-hour-distance standard requirement in the North and Central-west regions, where the average distance traveled, and the share of non-travelers are the highest [14]. Also, high-complexity centers are scarce, and small hospitals do not offer comprehensive emergency care [15]. Conversely, the average distance traveled is shorter in the Coastal Northeast, Southeast, and South regions, and traveling for childbirth is common. This is likely due to the proximity to capital cities, where high-complexity facilities are available, and mothers can access better care [15].

From 2007 to 2017, the distance traveled by pregnant women to access delivery services increased. The share of pregnant women traveling between municipalities rose from 23 to 31%, while the average distance traveled increased from 48 to 59 kilometers. Whether these changes have a positive impact depends on various factors related to accessing healthcare. Some studies have found that access to basic maternal and neonatal care is critical for the survival of newborns [32–36]. Hence, being closer to these services could positively affect the health of newborns. On the other hand, increasing the travel distance could improve access to more advanced or higher-quality facilities, which is also critical for newborn survival, especially for high-risk cases [37–39]. Moreover, health service locations should also respond to cost-effectiveness criteria. Concentrating health services in specific areas may be beneficial if it enhances service quality and reduces costs, even if it means increasing the distance to larger facilities [40].

Determining if the increase in travel distance has positive or negative implications for newborns’ health in Brazil goes beyond the scope of this paper. Nonetheless, our results suggest that the changes in distance could be attributed to improvements in the organization of the healthcare system and might be advantageous for newborns. Our study revealed that cases with risk factors tend to travel longer distances. Additionally, pregnant women from small-sized municipalities tend to travel longer distances, while those from medium-sized municipalities travel more frequently, albeit for shorter distances. Both these findings indicate that there is a concentration of births in specific locations, aligning with the regionalization process in Brazil, where there has been a significant increase in movements across municipalities for hospital care in recent decades [31]. Furthermore, another study from our research project found that the number of middle and high-complex facilities increased in Brazil between 2007 and 2017, and the share of high-risk births assisted in those facilities increased as well [41]. Overall, the evidence suggests that the distance increase is related to improved access to more comprehensive care.

Although some regions increased the number of women reaching other municipalities for childbirth, access to these services has not improved equally throughout the country. In northern states like Amazonas (AM), women travel more than 140 km to reach a health facility for childbirth, which has remained unchanged over the decade. This average distance equates to a journey of more than two hours, which is above the recommended time for addressing surgical emergencies [42]. Moreover, women traveling from northern states for childbirth have a high likelihood of surpassing their health regions, close to 50%. This highlights the need for better coordination of the healthcare system to ensure women receive appropriate care within the region’s limits. Despite some localized progress, persistent regional inequalities in travel distance to delivery facilities remain a concern.

The distance women travel for childbirth varies based on individual risk factors and social determinants. We find that those with high-risk pregnancies or premature, low-weight, low-APGAR newborns tend to travel further. One reason for traveling longer distances could be to access high-quality facilities that can handle obstetric and neonatal emergencies [33, 43, 44]. On the other hand, the average travel distance for childbirth is not affected by the number of prenatal visits a woman has, despite the high coverage of prenatal visits [12]. Prenatal visits should guide where to give birth and other health recommendations. Depending on individual needs, traveling for childbirth may be recommended. Unfortunately, inadequate prenatal care can lead to misinformation, increasing traveling distances unnecessarily [12].

Our results show a correlation between a locality’s living conditions and the distance mothers travel to give birth. In areas with higher socioeconomic development, mothers travel shorter distances. This suggests that there is a health inequity in the accessibility of delivery services, as all mothers should ideally have equal access to services regardless of their place of residence [45]. Similar socioeconomic gradients have been observed in other studies conducted in developing countries with respect to access to perinatal health services [46–48]. These results highlight the importance of taking into account the commonality between geography and socioeconomic health determinants when analyzing accessibility issues.

The study is limited by the data. We have analyzed and described travel patterns to access delivery facilities across Brazil for over a decade, but to do so, we make some simplifications. Specifically, we do not estimate the distance from the address of the pregnant woman to the delivery facility; instead, we use inter-municipal distances. Thus, we may have a downward bias in the estimated distances. However, it is reassuring for us that the geographic patterns discovered in the study are consistent with that of other Brazilian studies [14, 15], validating our results, at least from a macro perspective.

## Conclusion

This study analyses trends and regional inequalities in the distance women travel to access delivery services in Brazil between 2007 and 2017. We focus on two variables: the share of pregnant women leaving the residence municipality for childbirth and the average distance they travel. We find striking regional differences in shares and average travel distances to delivery services. In northern and inland states, average travel distances are long, but the share of pregnant women traveling is small. On the contrary, in coastal and southern states, the average travel distance is shorter, but the share of travelers is greater. Over time, the share of travelers and the average travel distance increased, and in spite of some local progress, regional inequalities persisted.

The distance women travel for childbirth varies depending on individual risk factors and social determinants. High-risk pregnancies and newborns at risk travel longer distances, which would be expected if specialized centers were farther apart. More importantly, the socioeconomic conditions of the pregnant woman’s place of residence are also associated with the distance she travels. Women from municipalities with higher socioeconomic development travel shorter distances. From a policy point of view, all pregnant women should have access to similar services regardless of their residence. The differences in travel distance by place of residence indicate potential social inequalities that should be addressed.

It is crucial to comprehend the factors that influence the distance between pregnant women and perinatal health services, as it significantly affects neonatal mortality rates [37–39]. In particular, we have identified several risk factors and social determinants that may affect travel distances. Additionally, we have found that using multiple alternatives to measure geographic access, such as shares and trip lengths, can help us better understand pregnant women’s movements to access delivery services. Hence, we believe that further research into how these alternative measures are affected by multiple factors could lead to a better understanding of health accessibility issues.

## Data Availability

The data used in this study combines information from the Brazilian Information System of Live Births [26], available from: https://pcdas.icict.fiocruz.br, and Carvalho et al.’s inter-municipal distance matrix [27], from https://EconPapers.repec.org/RePEc:cdp:texdis:td630. Data in Figs. 1-9 was created by aggregating this birth-distance dataset at the state, municipal, and national levels. Figures 1 and 2’s shapefiles come from Ref. [49], from https://www.ibge.gov.br/geociencias/organizacao-do-territorio/malhas-territoriais/15774-malhas.html?edicao=27733&t=acesso-ao-produto., and Figs. 3-8’s shapefiles come from Ref. [50], from https://github.com/lansaviniec/shapefile_das_regionais_de_saude_sus. Data in Figure 10 combines the birth-distance dataset aggregated by municipality with census data from Ref. [29], from http://www.atlasbrasil.org.br.

## Appendix

**Table 2:** This table estimates the correlations of socioeconomic variables listed in Figure 10 on distance, in a multivariate regression. Variables whose correlation with any other was above 0.9 were removed from regression to avoid redundancy. Clustered-robust standard errors are in parentheses at the health region level, only in column (2). Weight matrix for Moran’s I was extracted from [27] by reshaping and inverting their data. *** *p*<0.01, ** *p*<0.05, * *p*<0.1

	*Dependent variable:*	
	Distance	
	(1)	(2)
Rural population share	4.807$$^{***}$$	4.807$$^{***}$$
	(0.474)	(0.627)
Fertility rate	4.815$$^{***}$$	4.815$$^{***}$$
	(0.521)	(1.046)
Illiteracy rate (18 years +)	5.241$$^{***}$$	5.241$$^{***}$$
	(0.902)	(1.456)
Gini index	−1.276$$^{***}$$	−1.276$$^{**}$$
	(0.466)	(0.634)
Employment rate (18 years +)	−2.462$$^{***}$$	−2.462$$^{***}$$
	(0.468)	(0.627)
Population rate with inadequate water supply	6.558$$^{***}$$	6.558$$^{***}$$
	(1.142)	(2.048)
Population rate with electric power	−4.461$$^{***}$$	−4.461$$^{***}$$
	(0.497)	(1.132)
Population rate with bathroom and water	−3.114$$^{***}$$	−3.114$$^{**}$$
	(0.795)	(1.542)
HDI Education	−2.200$$^{***}$$	−2.200$$^{**}$$
	(0.730)	(1.085)
HDI Longevity	0.482	0.482
	(0.710)	(0.916)
HDI Income	−2.714$$^{**}$$	−2.714
	(1.071)	(1.655)
Observations	5,565	5,565
Adjusted R$^{2}$	0.187	0.187
Moran’s I	0.035	0.035
Moran’s I *p*-value	0	0
Cluster	No	Health Region

**Table 3:** State’s name and abbreviation

State	Abbreviation
Rondônia	RO
Acre	AC
Amazonas	AM
Roraima	RR
Pará	PA
Amapá	AP
Tocantins	TO
Maranhão	MA
Piauí	PI
Ceará	CE
Rio Grande do Norte	RN
Paraíba	PB
Pernambuco	PE
Alagoas	AL
Sergipe	SE
Bahia	BA
Minas Gerais	MG
Espírito Santo	ES
Rio de Janeiro	RJ
São Paulo	SP
Paraná	PR
Santa Catarina	SC
Rio Grande do Sul	RS
Mato Grosso do Sul	MS
Mato Grosso	MT
Goiás	GO
Distrito Federal	DF

## Abbreviations

SINASC: Sistema de Informações sobre Nascidos Vivo
SUS: Sistema Único de Saúde
ICU: Intensive Care Unit
NICU: Neonatal Intensive Care Unit
PCDas: Plataforma de Ciência de Dados aplicada á Saúde
DATASUS: Data Processing Department of SUS
ETL: extract, transform, load
OSM: Open Street Map
APGAR: Appearance, Pulse, Grimace, Activity, Respiration
HDI: Human Development Index

## Footnotes

1 We chose this analysis period based on data availability. First, we linked birth and mortality records from Brazil at the individual level. The identification code for these matches is available until 2017, so our analysis cannot cover more recent years. Subsequently, we included the distance each pregnant woman traveled for childbirth from the municipality of origin to the municipality of destination. Finally, we also included characteristics of the childbirth facility, such as complexity level, human resources, and infrastructure, utilizing data from the National Census of Health Facilities (CNES in Portuguese). This data source provides robust coverage only after 2007.

2 “F” is measured in a percentage and calculated as follows: $NPT_{it} / TP_{it}$, where $NPT_{it}$ represents the number of pregnant women who traveled and $TP_{it}$ the total number of pregnant women in the region *i* and time *t*.

3 “C” is measured as the mean of the distance traveled, considering the sample size of the pregnant women who have traveled in the region *i* and time *t*.

4 APGAR Index is a classical measure of newborn’s condition at first or fifth minutes of life based on heart rate, respiratory effort, reflex irritability, muscle tone and color. Maximum rate by criteria is 2, hence APGAR ranges from 0 to 10 and scoring lower than 8 means being rated 0 in one out of five criteria or being rated 1 in two criteria. 8+ is considered excellent prognosis and we used lower than 8 to align with other measures, since they indicate more risk for adverse health outcomes [30].

5 Since this could be a source of bias, we test a multivariate regression including just variables whose correlation with any other is below 0.9 and present results in Appendix Table A1, with no change in qualitative findings. Dependent variable distance is inherently spatial and Moran’s I rejects no autocorrelation null hypothesis, as expected for a measure such as this.
